# Roles of Cannabinoids in Melanoma: Evidence from In Vivo Studies

**DOI:** 10.3390/ijms21176040

**Published:** 2020-08-21

**Authors:** Ava Bachari, Terrence J. Piva, Seyed Alireza Salami, Negar Jamshidi, Nitin Mantri

**Affiliations:** 1 School of Science, RMIT University, Melbourne, VIC 3083, Australia; Ava.Bachari@student.rmit.edu.au; 2 School of Health and Biomedical Sciences, RMIT University, PO Box 71, Melbourne, VIC 3083, Australia; terry.piva@rmit.edu.au (T.J.P.); negar.jamshidi@rmit.edu.au (N.J.); 3 Faculty of Agricultural Science and Engineering, University of Tehran, Karaj 31587, Iran; asalami@ut.ac.ir

**Keywords:** medicinal herbs, apoptosis, THC, CBD, melanoma, cannabinoids

## Abstract

Melanoma is the fourth most common type of cancer diagnosed in Australians after breast, prostate, and colorectal cancers. While there has been substantial progress in the treatment of cancer in general, malignant melanoma, in particular, is resistant to existing medical therapies requiring an urgent need to develop effective treatments with lesser side effects. Several studies have shown that “cannabinoids”, the major compounds of the *Cannabis sativa* *L.* plant, can reduce cell proliferation and induce apoptosis in melanoma cells. Despite prohibited use of *Cannabis* in most parts of the world, in recent years there have been renewed interests in exploiting the beneficial health effects of the *Cannabis* plant-derived compounds. Therefore, the aim of this study was in the first instance to review the evidence from in vivo studies on the effects of cannabinoids on melanoma. Systematic searches were carried out in PubMed, Embase, Scopus, and ProQuest Central databases for relevant articles published from inception. From a total of 622 potential studies, six in vivo studies assessing the use of cannabinoids for treatment of melanoma were deemed eligible for the final analysis. The findings revealed cannabinoids, individually or combined, reduced tumor growth and promoted apoptosis and autophagy in melanoma cells. Further preclinical and animal studies are required to determine the underlying mechanisms of cannabinoids-mediated inhibition of cancer-signaling pathways. Well-structured, randomized clinical studies on cannabinoid use in melanoma patients would also be required prior to cannabinoids becoming a viable and recognized therapeutic option for melanoma treatment in patients.

## 1. Introduction

The history of humans and pathogens combat back to many years ago. Some other diseases, such as different types of cancers including melanoma, later emerged, and are mostly related to human genetics backgrounds and one’s lifestyle. However, it is quite difficult to say who the winner is; nobody can ignore the crucial role of traditional medicine and modern medicine in overcoming the disease’s attack. Regardless of accessibility, availability, and affordability, both have saved millions of lives. However, it can be said that herbal medicine has been associated with life since the beginning of human existence. Traditional medicine is defined as the sum total of the knowledge, skills, and practices indigenous to different cultures, used in the maintenance of health, as well as in the prevention, diagnosis, improvement, or treatment of physical and mental illness (World Health Organization). In this way, medicinal herbs as fresh or dried raw materials composed of crude raw plant material, standardized plant extracts, and isolated pure compound molecules are the basic pillar of traditional medicine [1].

The use of natural materials originating from medicinal herbs and replacing synthetic chemicals with these natural products is one of the most important needs of today’s civilization, especially in developing countries [2]. Among 35,000–70,000 medicinal plant species, some are more famous for cancers. There is a growing number of articles that explore the importance of compounds called “cannabinoids” exclusively found in cannabis plants, which can reduce cell proliferation and induce apoptosis in melanoma cells.

Melanoma is a highly metastatic skin cancer whose global incidence and mortality continues to rise, with about 80% of skin cancer-related deaths associated with melanoma [3]. In 2018, approximately 278,723 new cases of melanoma resulting in an estimated 60,712 deaths were reported in 46 countries [4]. The incidence and mortality rates were higher in males (150,698 and 34,831, respectively) than in females (137,025 and 25,881, respectively) in these countries [4]. According to the Australian Institute of Health and Welfare, as of 2019, melanoma is the third most common cancer in both females and males, with an incidence rate that is even higher than that of lung cancer. A recent study analyzed the standardized data on melanoma incidence rates (up to 2015) in susceptible populations in Canada, the United States of America, Australia, Denmark, Sweden, Norway, the United Kingdom, and New Zealand [5]. This study showed that Australia had the highest rate of occurrence, with 50.3 people for every 100,000 people, followed by New Zealand (47.4 in every 100,000), Denmark (32.7 in every 100,000), and Canada (17.9 in every 100,000) had the lowest incidence rate [5]. Further analysis according to the Australian Institute of Health and Welfare showed that as of 2019, the estimated number of new cases and death was 15,229 and 1725 in melanoma, respectively, which was higher than those from previous years. This data indicate that melanoma is a significant health risk in Australia. The main factors associated with the development of melanoma include exposure to UV rays [6], age, and male gender [7], individuals with a family history of skin cancer [8], and poor immune function or rare genetic abnormalities [9].

For more than three decades, the major chemotherapeutic agents for melanoma therapy have been combination of cisplatin and 5- fluorouracil which act selectively, promoting apoptosis by interfering with DNA synthesis in actively dividing cancer cells [10]. Later on, a cytostatic alkylating agent was introduced as a standard option for chemotherapy in clinical management of melanoma [11]. Temozolomide and dacarbazine were used in particular for treatment of early non-metastatic melanoma, but the general success was very limited for metastatic melanoma [12]. Numerous mutant BRAF (v-raf murine sarcoma viral oncogene homolog B1) inhibitors have been developed to target these mutant proteins. Originally, the most effective inhibitor was PLX4032 (also known as vemurafenib) with a 69% response rate in phase 1 clinical trials [13,14]. Preclinical studies further showed that antibodies against CTLA4 (T-lymphocyte-associated protein 4) induced regression of some murine tumors. As a result, currently two CTLA4 blocking monoclonal antibodies have entered pivotal clinical trial testing [15]. Ipilimumab (MDX010) is an antibody that targets human CLTA4 protein, and enhances T-cell activation and proliferation including the tumor-infiltration T-effector cells [16]. Tremelimumab is another antibody inhibitor for CTLA4 by 6.6% response rate in phase II of trial testing in metastatic melanoma [17]. Nivolumab is a human monoclonal antibody that antagonizes the programmed cell death protein-1 (PD-1) and PD-2 by blocking their receptors [18]. Thus, antibodies targeting CTLA4 and the PD-1 appear particularly effective targeted immunotherapies for melanoma and as the underlying mechanisms are unraveled, these inhibitors may be combined with alternative drugs such as cannabinoids to improve anti-tumor immune responses of patients with advanced melanoma or those responding to the current therapies.

The efficacy of all these conventional melanoma therapies such as surgical resection, chemotherapy, and immunotherapy is limited due to the high metastatic rate of melanoma and multiple resistance mechanisms coupled with substantial undesirable side effects of some of these therapies [19]. Developing new methods and therapeutic strategies to treat this aggressive cancer is therefore critical. The human skin has the endocannabinoid system composed of enzymes, receptors, and ligands which regulates skin homeostasis including the release of inflammatory compounds, cell differentiation, and division [20]. This system does have receptors for multiple compounds, including those derived from plants such as cannabis/hemp.

Cannabinoids are important compounds exclusively derived from the plant *Cannabis sativa* L. which could be potential agents for the treatment of melanoma. There are more than 120 known phytocannabinoids that can be found from *Cannabis sativa* L., cannabidiol (CBD) and tetrahydrocannabinol (THC) are the most abundant cannabinoids originating from cannabis. Both of these cannabinoids act together with the cannabinoids system and cause various natural effects [21]. Tetrahydrocannabinol (THC) can bind to receptors in the endocannabinoid system, helping to regulate cell division and potentially inhibiting or killing melanoma [22]. Therefore, cannabinoids have been used as therapeutic agents for several human and animal disorders including cancer [23,24,25].

The use of cannabis has always been very controversial because it is classified as an illegal drug due to THC. Despite this classification, there has been an increased scientific interest in recent years of the potential use of cannabis derivatives in medical applications [26]. Review of recent work has indicated that targeting the endocannabinoid system with cannabinoids can reduce the growth of breast, colon, liver, and prostate cancer [22,27]. Cannabinoids have also been used to successfully treat cancer cachexia, increasing the appetite of cancer patients; however, the associated side effects unfortunately adversely affected the patients’ quality of life [28]. Cannabinoids do not just stimulate appetites in some patients but have also been used to reduce pain and nausea in cancer patients [27].

Medical cannabis and its derivatives can selectively target tumor cells without exerting a cytotoxic effect on healthy cells [29]. This is its main benefit compared to chemotherapeutic agents which also affect cancerous tissues [30]. Extracts from *Cannabis sativa* L. have the potential not only to enhance survival rates but also to potentially improve the quality of life in melanoma patients [31]. While a great number of in vitro studies [32] (see Glodde et al. (2015)) have provided evidence of positive outcomes on using cannabinoids for treating melanoma cells, there are few in vivo and clinical studies that have been published [33,34].

The mechanism of cannabinoid action is associated with G-protein coupling with cannabinoid receptors CB1 and CB2. The CB1 receptor is present mostly in the brain or on the membrane of nerve cells. It is believed that the psycho-activity triggered by cannabinoids is largely controlled by this receptor. CB2 receptors are expressed only in immune cells, such as T and B lymphocytes and macrophage [35]. Through these interactions, cannabinoids regulate the signaling pathways involved in cell division, inhibiting division or metastasis of cancer cells, enhancing autophagy, and inducing apoptosis [36,37].

In this study, we searched in different databases such as SCOPUS, EMBASE, ProQuest Central, and PubMed from inception. Of the 622 screened studies, six studies with were included which matched our criteria. We assessed whether cannabinoid use was effective for treating melanoma by investigating tumor growth, inhibition of metastasis, and quality of life and movement. The finding from this systematic review should show whether cannabinoids have been effective for melanoma treatment, and provide a basis for its clinical use.

## 2. Methods

### 2.1. Inclusion Criteria

In this review, the inclusion criteria for relevant studies were:

- The study should be a primary research article;
- The study should include the use of cannabinoids (endocannabinoid, phytocannabinoids, and synthetic);
- Cannabinoids should have been used in treating melanoma;
- In vivo studies that explored the effect of cannabinoids on tumor activity or size were selected;
- All study types were included except reviews and commentaries.

### 2.2. Exclusion Criteria

The exclusion criteria were:

- The article was not written in English;
- It was a judgement article;
- The reported study was not on animals;
- The article or study was not related to melanoma;
- The article was not related to cannabinoids.

### 2.3. Search Strategy for Identification of Studies

**Figure 1:**
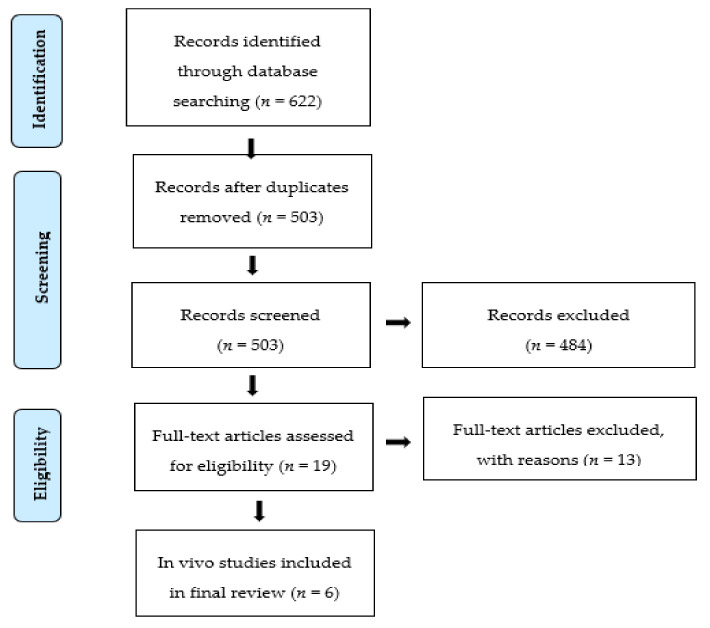
Flow diagram showing the search process and results used in this study.

In this review, the PRISMA (preferred reporting items for systematic reviews and meta-analyses) guidelines and checklist were followed [38]. Comprehensive literature searches were conducted using the EMBASE, SCOPUS, ProQuest Central, and PUBMED databases. The first step involved using “cannabis” and “cannabis-related”, “melanoma”, “in vivo”, or “animal” as search terms. A combination of these key terms was used to generate other search terms such as Cannabinoids* OR “*Cannabis sativa*” OR Tetrahydrocannabinol OR “THC” OR cannabidiol OR CBD OR “cannabidiol acid“ OR CBDC OR cannabigerol OR CBG OR “cannabis chromene” OR CBC OR anandamide OR AEA OR “endocannabinoids” OR “2-AG” OR “Arachidonoyl glycerol” OR “HU-210” OR “WIN-55” OR “JWH-015” OR Methanandamide OR “JWH-133”AND Melanoma OR “skin cancer” OR “malignant melanoma” OR melanocarcinoma OR melano-epithelioma OR melanosarcoma AND in vivo OR ex vivo OR animal OR experimental OR xenograft. The reference lists of relevant studies were also checked. Two reviewers (A.B. and N.J.) reviewed the abstracts and full texts independently for inclusions and exclusions. Any differences in inclusion or exclusion were determined by consensus or consultation with a third reviewer (N.M.). The studies and the relevant data were classified and extracted in accordance with the assessed procedure. The titles and abstracts of retrieved articles were then screened in order to select relevant articles for inclusion. Full-text screenings were performed, and relevant data were extracted from these eligible studies. The detailed process of the search as a PRISMA flow chart is presented in Figure 1.

### 2.4. Data Extraction and Synthesis

In total, 622 titles and abstracts (using the inclusion and exclusion criteria) were screened according to the eligibility criteria for inclusion in this study. There were 503 non-duplicated articles detected during this screening of articles obtained via literature search. After screening, a total of 19 studies were selected and were further evaluated to determine which of them were in vivo or in vitro studies, with the latter being excluded. The inclusion/exclusion criteria and main characteristics of the six included studies are presented in Table 1. This table describes the key findings of this systematic review including the results of melanoma and cannabinoids interactions, studies on tumor growth in mice, description of the specific cannabinoid or receptor involved in the regulation of cell (tumor) proliferation in test subjects, and the corresponding supporting studies.

### 2.5. Quality Assessment

The risk of bias was assessed using SYRCLE’s (SYstematic Review Centre for Laboratory animal Experimentation) risk of bias tool for animal studies [39]. According to SYRCLE’s tool selection biases include random group allocation, baseline group allocation, allocation conceal; performance bias contains random hosing, blinding of examiner; detection bias comprises random outcome selection, blinding of assessor; attrition bias and other biases include any randomization, any blinding, size calculation, or temperature control. Two researchers independently screened the literature, extracted the data, and performed the cross-check. In the case of a disagreement, the resolution was reached through discussions with a third researcher.

### 2.6. Assessment of the Risk of Bias in the Included Studies

In this assessment “YES” indicated low risk and “NO” indicated high risk. The results obtained from a detailed analysis of the six selected studies showed that half of the included studies were classified as a low-risk random group. Half of these studies showed a high risk of the baseline group characteristic. All of these studies had a high risk of allocation conceal, and two of these included studies are at low risk of random housing. All of the studies were at high risk for blinding of an examiner, random outcome selection, blinding of assessor, and attrition bias. For other biases, half of the studies had any randomization and none of them was mentioned for any blinding, size calculation, or temperature (Table 2).

## 3. Results

The result of our search strategy was 622 articles initially identified through database searches. One hundred and nineteen duplicate studies were discarded, leaving 503 single studies. An additional 484 studies were removed after the title and abstract screening, excluding non-English articles, studies not addressing the research question, and expert opinion articles. Nineteen full-text articles were reviewed for eligibility for inclusion in the final analysis. Further evaluation showed that 40%, or six of these 19 records, were in vivo studies and were therefore included in this review. As the focus of this review was on in vivo studies, the in vitro studies were subsequently excluded (Figure 1).

### Cannabinoids and Melanoma Cancer

A few studies have evaluated the presence of cannabinoid receptors (CB1 and CB2) and the role of cannabinoids in carcinogenesis and cell proliferation of melanoma in vivo. As a result, Glodde et al. (2015) demonstrated that ∆9-THC significantly reduced the growth of HCmel12 melanomas in mice by about 50%, according to vehicle-treated mice after 25 days. However, this reduction was not observed in mice without the CB1/CB2 receptors, indicating the importance of a CB receptor for the inhibition of metastasis and the possibility of this occurring via a CB receptor-dependent mechanism. This study also provided some new insights into the potential role of natural or synthetic CB receptor agonists in the treatment of cancer types characterized by a pro-tumorigenic inflammatory microenvironment. Armstrong et al. (2015) showed that cannabinoids (THC) induced autophagy in SK-MEL-28, A357, and CHL-1 melanoma cells, through an apoptotic-like mechanism. Co-treatment with cannabidiol (CBD) and ∆9-THC was also observed to exert a synergistic cytotoxic effect on these cells. Using a mixture of THC and the non-psychoactive cannabinoid CBD, a laboratory mimic of the clinical cannabinoid Sativex^®^ (an oromucosal spray) containing equal amounts of THC and CBD reduced glioma growth in vivo at the same level as an identical dose of THC [40]. The cannabinoid THC was found to exert its antitumor effect on melanoma cells via the activation of non-canonical autophagy and subsequent apoptosis in this study. Hamtiaux et al. (2012) investigated the possibility of enhancing endocannabinoid cytotoxicity using inhibitors of their hydrolysis in the melanoma model. The results of their investigations showed that the co-administration of PEA and URB597 caused a significant reduction in the growth of tumors and their sizes.

In another investigation, ∆9-THC activity resulted in the inhibition of the activation of pro-survival proteins, Akt and pRb, in melanoma compared to the non-tumorigenic melanocytes [41]. The activation of CB receptors with WIN-55,212-2 (synthetic cannabinoids) in vivo caused notable reductions in cell viability, growth of tumor, and metastasis. Another study showed that the systematic administration of ACEA, a stable CB1 agonist, to SCID mice inhibited liver colonization of human melanoma [42]. Simmerman et al. (2018) reported that the administration of cannabidiol repressed the tumor size significantly compared with the untreated group, while cisplatin demonstrated a greater reduction in tumor size but was associated with a lower quality of life (Table 1). Further research is needed to clarify the exact role and particular mechanisms exploring the endocannabinoid and receptors involved behind such a phenomenon.

## 4. Discussion

Marijuana is classified as an illicit drug and its use is prohibited in most countries. However, cannabis/hemp strains contain over 120 phytocannabinoids, including cannabidiol (CBD) and tetrahydrocannabinol (THC), which are thought to be of therapeutic more importance [22,31,43]. Apart from phytocannabinoids, other classes of cannabinoids such as endocannabinoids and chemically synthetic cannabinoids are known [44]. Cannabinoids have an extensive role in palliative care, which includes inhibition of nausea and emesis related to chemo- or radiotherapy, appetite stimulation, pain relief, mood elevation, and relief from insomnia for some oncology patients [45].

This systematic review was focused on the role of cannabinoids as antiproliferative agents in melanoma in studies carried out in vivo in recent literature. Several studies have shown that cannabinoids can reduce cell proliferation and induce apoptosis in melanoma cells [41,46]. While there is an abundance of literature related to the biological mechanisms for melanoma cancer and their interactions with cannabinoids in vitro, very limited studies have been carried out on the effects of cannabinoids on melanoma cancers in vivo and or evaluated with clinical trials. This is why only six papers were related to the desired in vivo parameters out of the over 622 papers that were screened. Despite the availability of limited information, scientific understandings of the therapeutic role of cannabinoids as antiproliferative and tumor-regression agents remain a significant research interest. The comparatively limited number of in vivo studies probably explains why there were fewer clinical trials on cannabinoid treatment of melanoma reported in peer-reviewed articles. This is because a successful in vivo test is a pre-requisite for clinical trials.

A review of literature has shown that multiple clinical trials have been carried out using CBD and THC to treat a variety of medical issues ranging from post-traumatic stress disorder (PTSD), chronic pain, to multiple sclerosis [47]. In some of the trials evaluated, the use of cannabinoids was found, in addition to alleviating the symptoms of the diseases being targeted, to also improve the quality of sleep in clinical subjects by reducing sleep disturbance episodes and reducing the onset of sleep latency [47]. Preliminary data from some of the clinical trials have indicated that the use of CBD can alleviate the symptoms of acute schizophrenia [48], and the use of THC and/or CBD is thought to potentially reduce chronic pain in some patients and may require further trials for confirmation [49]. For example, THC/CBD extracts have been shown to provide pain relief in some patients with advanced cancer whose pains had not been successfully relieved by opioid pain killers [50]. However, a different review of clinical evidence on the relief of cancer-related pain showed conflicting results, and the authors suggest that there may be limited or no significant evidence of cannabinoids causing a demonstrable reduction in cancer pain [51]. Other clinical trials such as the CUPID (Cannabinoid Use in Progressive Inflammatory brain Disease) trial on the use of cannabinoids for the treatment of progressive inflammatory brain disease have shown no beneficial effect of cannabinoid use on disease progression [52].

With respect to cancer, there are limited but hopeful clinical trials relating to the role of cannabinoids for the treatment of malignancies. A study in Israel is studying the efficacy of the use of cannabinoids as a treatment in patients with tumors that are resistant to chemotherapy (NCT02255292) [53]. Another study is a phase 1/2 trial that is assessing the combined effect of Sativex^®^ and temozolomide in patients with recurrent glioblastoma multiforme (NCT01812603 and NCT01812616) [54,55]. Other small clinical trials have shown some regression in tumor sizes associated with the use of cannabinoids or cannabinoid extracts [55].

Out of the six papers on in vivo studies selected in this review, four of them demonstrated the beneficial effects of cannabinoids against melanoma. It is important to note in most of these studies, in vitro assays were initially carried out to validate that the selected treatments had reduced tumor-genesis and proliferation in melanoma cell lines. Firstly, evidence from some of the selected in vivo studies showed that cannabinoids can be used individually to successfully reduce tumor sizes and induce cell death. Two of the selected studies clearly demonstrated this; the use of THC in mice with induced BRAF/NRAS wild-type tumors promoted autophagy in melanoma cells [32]. Similarly, the use of CBD on mice with an induced B16F110 tumor significantly reduced tumorigenicity and tumor size [31].

Secondly, in some instances, the use of cannabinoids can potentially improve the quality of life of cancer patients. When compared to an equally effective non-cannabinoid compound cisplatin, CBD-treated mice had improved movement in and out of the cage and less hostile interactions compared to cisplatin [31]. This behavioral improvement could translate to a potentially better quality of life (less agitation and stress) in some human subjects with melanoma.

Thirdly, using a combination of cannabinoids can also be effective in melanoma treatment. The THC/CBD combination (Sativex^®^) substantially promoted autophagy, apoptosis, and loss of viability in melanoma cells compared to a non-cannabinoid compound, temozolomide [32]. Sometimes, a better treatment effect as a result of synergy was observed, with THC/CBD causing a substantial loss of melanoma viability compared to THC alone [32].

Fourthly, these reductions in tumor size, enhancement of autophagy, and apoptosis can occur in the presence or absence of CB1 and CB2 cannabinoid receptors. Activation of these receptors by cannabinoids and other agonists reduced cell proliferation and metastasis, and promoted the death of cancer cells in in vivo studies [41]. However, in the absence of these receptors, a similar result was observed. In wild-type (WT) and CB1/CB2 receptor-deficient mice, substantial inhibition of tumor growth (skin cancer cells) in both types of mice was observed when THC was used for melanoma treatment [56]. This indicated that cannabinoids can reduce melanoma growth by other mechanisms that do not involve the use of endocannabinoid receptors.

The epidermal layers of the skin are made up of different types of cells such as keratinocytes and melanocytes which are the source of malignant and non-malignant skin cancer [55]. In addition to these cells, the ECS of the skin has also been implicated in skin cancer development, regulation, and control with CB1 and CB2 receptors thought to play important roles based on their interactions with cannabinoids and endocannabinoids [55]. Reviews of past work by Velasco et al. (2015) have shown that cannabinoid anti-tumor activities occur via the induction of cancer cell death, inhibition of the spread of cancer cells, and the promotion of immune activities that suppress tumor [57].

A number of studies support the notion that cannabinoids could potentially enhance the immune response, thereby preventing growth and spread of tumors. An in vivo melanoma xenograft model showed that the activity of WIN 55,212-2 promotes tumor regression efficiently in the immunocompetent mice against immunodeficiency [41,42,43,44,45,46,47,48,49,50,51,52,53,54,55,56,57,58]. Another study showed that CBD, THC, and R(+)-methanandamide make lung cancer cell susceptible to lysis by lymphokine killer cells [59]. The underlying mechanism is thought to include upregulation of ICAM-1 (intercellular adhesion molecule 1) on the surface of cancer cells by cannabinoids leading to crosslink with the related lymphocyte function antigen-1 on the surface of killer cells. Moreover, another study indicated that infiltration reduction for macrophages and neutrophils in animals treated with THC of experimental skin lead to tumor regression [56,58]. Therefore, cannabinoids may facilitate an antitumor immune response through independent mechanisms by triggering a more responsive immune system to combat cancer or provide favorable conditions such as localized reduction in pro-carcinogenic inflammatory microenvironment within the cancer tissue. However, a previous study showed that THC accelerated the differentiation of breast cancer cells as a consequence of inhibiting the anti-tumor immune response [60]. Hence, further research is required to elucidate the role of cannabinoids in tumor progression and immune interactions within the cancer tissue. More recently, there are a substantial surge in the use of immunotherapy and cannabinoids. A recent study showed that combination of cannabis and nivolumab as an immunotherapy agent for patients with advanced malignancies decreased the response rate, and did not affect the progression-free survival or overall survival, and without relation to cannabis composition [61].

CB1 and CB2 cannabinoid receptor-stimulation by cannabinoid agonists can lead to the apoptosis of cancer cells. This occurs because the stimulation of the receptors leads to the synthesis and release of a compound called ceramide. Ceramide synthesis stimulates the production of endoplasmic reticulum stress-related factors although cannabinoids such as THC upregulate stress-regulated proteins such as p8 or NUPR1. These stress-proteins can regulate (inhibit) tumor generation and production alongside other transcriptional factors such as endoplasmic reticulum-ATF4, TRIB3, and CHOP, and enhance autophagy [57]. Cancer regulation can also occur independently of CB1 and CB2 receptors [62].

In conclusion, evidence from these in vivo studies suggest that the use of THC and CBD not only inhibited tumor growth and reduced tumor size but also seemed to improve the quality of life in animal models [31]. A synergistic approach (using two cannabinoids in combination) may be more beneficial for melanoma treatment than the use of individual cannabinoids [32] with a potentially improved quality of life in some patients. Therefore, future in vivo studies should include both individual cannabinoid and combined cannabinoid-based approaches for the treatment of melanoma and the investigation of the mechanism underpinning the synergistic effects observed. In addition, given the large number of in vitro studies, future reviews will be needed to identify the potential underlying mechanisms of cannabinoids involved in the inhibition of melanoma and to contribute further to our understanding of the complex endocannabinoid system involvement in the treatment of cancer.

## Figures and Tables

**Table 1:** Summary of eligible studies comparing the effects of cannabinoids on melanoma.

First Author (Year)	Study Population (Animals)			Tumor Induction (Cell Line)	Study Intervention		Dose of Cannabinoid	Duration	Anticancer Outcomes
	Strain	Age	Number		Intervention	Control			
Hamtiaux 2012	C57BL/6mice	5–weeks	*n* = 6	B16 melanoma cells	PEA+URB597 (s.c)	Vehicle	10 mg/Kg	6 days	Co-administration of PEA and URB597 resulted in a significant reduction of tumor growth & size
Glodde 2015	C57BL/6mice Wild-type and CB1/CB2-deficient mice	8–10 weeks	*n* = 10	B16 melanoma cells HCmel12	THC (s.c)	Vehicle	5 mg/Kg per day	25 days	Inhibits HCmel12 melanoma growth but does not affect B16 and CB1/CB2 deficient HCmel12
Armstrong et al. 2015	Athymic nude mice	5 weeks	20 mice (*n* = 5 per group)	Xenograft CHL-1 cells	THC (oral) THC-BDS + CBD-BDS (oral) Temozolomide	Vehicle	15 mg/Kg (daily) 7.5 mg/Kg + 7.5 mg/Kg (daily) 5 mg/Kg (daily)	20 days	Reduction in tumor size THC-BDS + CBD-BDS ≥ THC > TEMO
Blazquez 2006	C57BL/6mice Nude mice		*n* = 8 (per group) *n* = 6 for each experimental group	B16 melanoma cells	WIN55-212-2(s.c) or JWH-133 (s.c) WIN55-212-2 (s.c)	Vehicle	50 mg/day 50 mg/day 50 mg/per3days	8 days 21 days	WIN55-212-2 = JWH-133 in preventing tumor growth Decreased tumor growth and metastasis
Kenessey 2012	SCID mice		*n* = 8	HT168-M1	ACEA (i.p)	Solvent control	0.24 mg/Kg 1/2 mg/Kg	21 days	CB1 agonistic AECA into SCID mice inhibit liver colonization of human melanoma cells
Simmerman 2018	C57BL/6mice	8–12 weeks of age)	18 (*n* = 6 per group)	Murine melanoma cell line, B16F10	CBD (i.p) Cisplatin (i.p)	Vehicle	5 mg/Kg twice per week 5mg/Kg once per week		Increased the quality of life and movement; significantly decreased growth curve and increased survival curve

**Abbreviations:** s.c (subcutaneously), i.p (intraperitoneally), BDS (botanic drug substance), CBD (cannabidiol), TEMO (temozolomide: Chemotherapy drug), WIN212-2 and JWH-133 (synthetic cannabinoids), URB597 (inhibitor of the enzyme fatty acid amide hydrolase), PEA (N-Palmitoylethanolamide), THC (Δ⁹-tetrahydrocannabinol), ACEA (synthetic cannabinoid), cisplatin (chemotherapy medication).

**Table 2:** Risk of bias assessment in animal studies using SYRCLE (SYstematic Review Centre for Laboratory animal Experimentation) tool *.

Study	Selection Bias			Performance Bias		Detection Bias		Attrition Bias	Other Bias			
	Random Group Allocation	Baseline Group Characteristics	Allocation Conceal	Random Hosing	Blinding of Examiner	Random Outcome Selection	Blinding of Assessor		Any Randomization	Any Blinding	Size Calculation	Temp Control
Armstrong 2015	L	H	H	H	H	H	H	H	Y	N	N	N
Blazquez 2006	L	L	H	H	H	H	H	H	Y	N	N	N
Hamtiaux 2012	L	L	H	H	H	H	H	H	Y	N	N	N
Glodde 2015	H	L	H	H	H	H	H	H	N	N	N	N
Kenessey 2012	H	H	H	L	H	H	H	H	N	N	N	N
Simmerman 2018	H	H	H	L	H	H	H	H	N	N	N	N

* H = High Risk, L = Low Risk, Y = Clear, N = Not Clear.
